# Radiotherapy Increases aMMP-8-Levels and Neutrophil/Lymphocyte Ratio Rapidly in Head and Neck Cancer Patients: A Pilot Study

**DOI:** 10.1177/10732748231163653

**Published:** 2023-04-24

**Authors:** Ella Brandt, Mutlu Keskin, Taina Tervahartiala, Mustafa Yılmaz, İlknur Harmankaya, Didem Karaçetin, Turgut İpek, Ulvi Kahraman Gürsoy, Jaana Rautava, Shipra Gupta, Jaana Hagström, Ismo T. Räisänen, Timo Sorsa

**Affiliations:** 1 Department of Oral and Maxillofacial Diseases, 3835 University of Helsinki and Helsinki University Hospital, Helsinki, Finland; 2 Oral and Dental Health Department, Altınbaş University, Turkey; 3 Department of Periodontology, Faculty of Dentistry, Biruni University, Istanbul, Turkey; 4 Department of Radiation Oncology, Başakşehir Çam and Sakura City Hospital, Istanbul, Turkey; 5 Department of General Surgery, Faculty of Medicine, Altınbaş University, Istanbul, Turkey; 6 Department of Periodontology, Institute of Dentistry, 8058 University of Turku, Turku, Finland; 7 Department of Oral Pathology and Radiology, 8058 University of Turku, Turku, Finland; 8 Unit of Periodontology, Oral Health Sciences Centre, 29751 Post Graduate Institute of Medical Education and Research, Chandigarh 160012, India; 9 Department of Pathology, Helsinki University Hospital and HUSLAB, Helsinki; 10 Department of Medicine and Dental Medicine, Karolinska Institutet, Stockholm, Sweden

**Keywords:** head and neck cancer, aMMP-8, radiotherapy, neutrophil/lymphocyte ratio, point of care technology, matrix metalloproteinases

## Abstract

Radiotherapy for head and neck carcinoma (HNC) has both curative and palliative purposes. This study investigated mouthrinse aMMP-8 levels, molecular forms of MMP-8, blood neutrophil counts and neurophil/lymphocyte ratios before and 3 weeks after HNC radiotherapy started. Thirteen HNC patients undergoing radiotherapy were included. Mouthrinse samples (before and 3 weeks after HNC radiotherapy had started) were assayed quantitatively by aMMP-8 point-of-care-kit (PerioSafe®/ORALyzer®) and by western immunoblot. Total neutrophil counts and neutrophil/lymphocyte ratios were evaluated in the hemogram results. Three weeks after HNC radiotherapy started, significant increases in aMMP-8 levels and neutrophil/lymphocyte ratios were observed. No significant difference was found in total neutrophil counts. Elevations of the activated and fragmented MMP-8 levels after HNC radiotherapy application were observed on western immunoblot analysis. The increase in the aMMP-8 levels and neutrophil/lymphocyte ratios indicate inflammation both locally and systemically suggesting increased risk for periodontitis due to the HNC radiotherapy.

## Introduction

Head and neck cancers include tumors occurring in the oral cavity, larynx, oropharynx, nasopharynx or salivary glands. Head and neck cancers are the seventh most common among all malignant tumors.^1^ The most commonly observed head and neck cancer is squamous cell carcinoma, which covers over 90% of these tumors.^2^ In addition to tobacco smoking and alcohol use, human papillomavirus (HPV) type 16 and Epstein-Barr virus (EBV) infections have been connected to the onset of HNC.^3^

The objective of any treatment strategy for HNC is to achieve the highest possible cure rate with the lowest risk of morbidity. As such, treatment proposals are determined by the stage and location of the primary lesion as well as specialist judgement and patient goals.^3^ The usual treatment-of-choice for head and neck carcinomas (HNC) are surgery and radiotherapy. They are used either separately or in combination depending on the tumor location, size and spreading.^4^ Lately, immune-oncological treatments have been accepted for HNC as well.^5^

In the treatment plans of HNC patients, radiotherapy is aimed to be performed for a 5-7-week period with a total dosage interval of 54–70 Gy. In some patients, chemotherapy can be given in combination to radiotherapy. Despite the multi-model approach, treatment success in HNCs has still not reached the desired level and the 5-year survival rate is under 65%.^6^ Radiotherapy aims to treat solely the tumor bed. However, it unfortunately has systemic side effects on areas apart from the target and can affect the success of eventual treatment.^7^ Immune cells, such as neutrophils and lymphocytes, are suggested to play role in this systemic effect.^8^

The neutrophil/lymphocyte ratio is of significant importance for the measurement of subclinical inflammation.^9,10^ Matrix metalloproteinase (MMP)-8 is a known neutrophil sourced collagenase or collagenase-2. MMP-8 is an important mediator in the pathogenesis in periodontal diseases.^11^ It is additionally associated with various systemic inflammatory conditions such as diabetes, pulmonary and cardiovascular diseases, as well as cancers. Studies have repeatedly shown that MMP-8 is the most concentrated collagenase found in saliva, mouthrinse and gingival crevicular fluid especially in patients with periodontal disease.^11,12^

MMPs, especially MMP-8, play a key role in tissue destruction processes that occur within the periodontal tissues infected by dystopic pathogenic microorganisms. This triggers the overall proinflammatory defense and tissue destructive mechanisms producing cytokines, reactive oxygen species and host derived proteolytic enzymes leading to periodontal disease.^13,14^

In this pilot study we wanted to find simple and accurate parameter for evaluating the risk of periodontal changes caused by HNC radiotherapy. Understanding of these phenomena could help us provide preventive and supportive actions and treatments to maintain the periodontal health of the patients. Worsening periodontitis may lead to a tooth extraction with the associated risk of osteoradionecrosis.

The aim of the present pilot study was to evaluate the rapid effect of the HNC radiotherapy on the blood neutrophil count, neutrophil/lymphocyte ratio, as indicators of systemic inflammation, and mouthrinse aMMP-8 levels as well as on molecular forms of MMP-8 among patients with HNC reflecting periodontal damage.

## Materials and Methods

### Patient Selection and Study Design

Thirteen patients from Başakşehir Çam and Sakura City Hospital, Istanbul, Turkey with histologically confirmed HNC were included in this prospective pilot study. However, additional retrospective data was included. Inclusion criteria were histologically confirmed head and neck carcinoma, at least 21 years of age; presence of ten teeth or more in the oral cavity and radiotherapy for HNC treatment. Exclusion criteria were: patients who had Eastern Cooperative Oncology Group (ECOG) performance of what 3 and higher; had immune associated disorders (i.e. chronic inflammatory diseases such as lupus erythematosus, rheumatoid arthritis, multiple sclerosis, Crohn’s disease); were on bisphosphonate therapy; or tested positive for human immunodeficiency virus (HIV).

Demographic data and the medical history including presence of systemic diseases, medications and smoking habits of the patients was recorded. Radiotherapy treatment was performed by radiation oncology expert physicians according to the stage level of the disease and following The National Comprehensive Cancer Network® (NCCN®) guidelines. Before radiotherapy periodontal health was examined by an experienced periodontist. Considering the systemic conditions, pathology and imaging reports of the patients, possible chemotherapy regimens were decided individually for each patient by expert medical oncologists being in line with the NCCN guidelines. Patients were instructed to maintain good mouth hygiene during HNC treatment.

This study has been approved by Biruni University Ethics Committee, Turkey (No: 2015-KAEK-53-21-05, date of approval 13.10.2021) and was conducted according to the World Medical Association Declaration of Helsinki. All individuals participating in the study signed the written informed consent form. Consent was titled “Comparative analysis of oral mmp-8 level with neutrophil and lymphocyte counts in patients with head and neck cancer treated with radiotherapy”. Patients were selected randomly. All the patient details have been de-identified so that the identity of any person may not be ascertained in any way. Relevant Equator guidelines were followed in this study. The reporting of this study conforms to STROBE guidelines.^15^

### The Periodontal Examination Procedure

A single periodontist (M.K.) performed baseline periodontal examination and determined the patients’ periodontal disease classifications as stages (I−IV) and grades (A−C) based on the 2017 Classification of Periodontal Disease^16^ by evaluating the extent of tooth support destruction, including clinical attachment level (distance between cemento-enamel junction and bottom of periodontal pocket), probing depths (distance between marginal gingiva to apical point of the gingival sulcus), pattern of bone loss, and more.

Stage I: Mild periodontitis with minimal attachment and bone loss.

Stage II: Moderate periodontitis with moderate attachment and bone loss.

Stage III: Severe periodontitis with significant attachment and bone loss.

Stage IV: Very severe periodontitis with very significant attachment and bone loss.

Grading was used to assess the risk for disease progression considering risk factors such as patient phenotype, smoking, and hyperglycemia.

Grade A: Slow progression rate.

Grade B: Moderate progression rate.

Grade C: Rapid progression rate.

### The Radiotherapy Procedure

The CT images of the patients were created as 3 mm thick sections with a CT simulator device (Toshiba Aquilian Computerized Tomography Toshiba®, Japan) using a thermoplastic head and neck mask. The CT images were transferred to the Monaco treatment planning system (CMS Inc, Version 5.1, St Louis, MO) for the contouring of all target volumes and critical structures in the head and neck area. Treatment plans were calculated using the Monaco treatment planning system.

Total radiotherapy treatment modality for each patient was planned as follows: For non-operative cases, a 70–72 Gy dosage was prescribed to the primary target, and a 54–58 Gy dosage was prescribed to the regional lymph nodes. For post-operative cases, the planned dosages for the primary tumor site were determined as 60–66 Gy. For all patients who underwent surgery, radiotherapy was performed post-operatively. All patients were treated with the IMRT technique using 6 MV photon energy and the Elekta Synergy Linear Accelerator (Elekta Oncology®, UK) device.

Radiotherapy was prescribed 6 weeks totally for each patient. Three weeks after radiotherapy started the patients arrived for a routine visit to the hospital. In a midway of the radiotherapy, the radiation received corresponded 30–35 Gy totally.

### The Collection of Oral Rinse/aMMP-8 Analysis

Mouthrinse sample was taken from each patient before radiotherapy and 3 weeks after radiotherapy started by a periodontist. Patients were instructed to avoid eating and tooth brushing an hour before the analysis. According to the protocol the patients first rinsed their mouths with tap water for 30 seconds and spit it out. After 1 minute of waiting, they were asked to rinse their mouth for 30 seconds with the 5 mL of the oral rinse solution provided by the kit followed by spitting the liquid into measuring cups.

The sample was taken from the measuring cup with a syringe and filtered with Millipore filters and transferred to the aMMP-8 Periosafe® kit test cassette (Dentognostics GmbH, Jena, Germany). The active form of matrix metalloproteinase-8 (aMMP-8) was quantitatively analyzed by the digital reader ORALyzer® according to the manufacturer’s instructions as described earlier.^17^ The overall sensitivity and specificity of the oral fluid aMMP-8 POCT-technologies for periodontitis and dental peri-implantitis are 70-80% and 80-95%, respectively.^17^

### Neutrophil and Lymphocyte Count Measurements

The venous blood samples taken from each patient were transferred into 5 mL tubes containing ethylenediaminetetraacetic acid (EDTA). Neutrophil and lymphocyte parameters were measured using a complete blood count device (BC-6800 Plus, Shenzen Mindray Bio-Medical Electronics Co., Nanshan 518057 Shenzhen, China) within 2 hours following blood collection.

### Western Immunoblot

The molecular forms of MMP-8 were detected by a modified enhanced chemiluminescence (ECL) western blotting kit according to protocol recommended by the manufacturer (Amersham ECL Western Blotting Detection Kit, Catalogue number RPN2108. Cytiva, Marlborough, MA USA) as described earlier.^18^ Briefly, the protein separation was performed by electrophoresis and the proteins were electrotransferred onto nitrocellulose Protran membranes (Whatman GmbH, Dassel, Germany). These membranes were incubated with polyclonal primary antibody anti-MMP-8 (dilution 1:500) overnight^18,19^ and then later with anti-rabbit IgG-horseradish peroxidase conjugated secondary antibody (1:350, Amersham ECL Western Blotting Detection Kit). The immunoblots were quantified by a Bio-Rad Model GS-700 Imaging Densitometer using Bio-Rad Quantity One program (Bio-Rad Laboratories Inc., Hercules, CA, USA).

### Statistical Analysis

The statistical evaluation of the changes in the parameters between two-time points (pre-radiotherapy and after 3 weeks of radiotherapy) were performed with the paired-samples t-test (2-tailed) using the SPSS for Mac OS 21 (SPSS Inc., Chicago, IL) program. P-values of <.05 were accepted as statistically significant. Although study group was small, we found significant results and it can be concluded that the power was large enough in this study.

## Results

### The Characteristics and Baseline Periodontal Status of the Patients

Patient, HNC and treatment characteristics are presented in Table 1. Patients (11 male, 2 female) were on an average 56 (SD = 13.5) years old at the time of the HNC diagnosis. Because of the small study group, the patients’ age is given in a scale rather than exact age in Table 1. Eight patients had additional conditions, the most common of which was chronic obstructive pulmonary disease. All patients had history of smoking (≥10 cigarettes per day) of more than 5 years but had quitted smoking just before chemoradiotherapy. Staging and grading of periodontitis for each patient is presented in the Table 2. In our study group, 6 patients were diagnosed with Stage IV periodontitis, 2 with Stage III and five with Stage II. All patients were diagnosed with Grade C periodontitis.

**Table 1.** The Characteristics of the Patients.

Patient	Sex	Age (years)	Additional Diseases	Medication	Histopathological dg	Tumor Location		T/N/M	Stage	Treatment	Dosage of RT (Gy)
1	M	60-79	None	None	SCC	Larynx	2/0/0		II	S + RT	31,7
2	M	60-79	Hypothyroidism, COPD	Levothyroxine sodium	SCC	Larynx	3/0/0		III	RT	33,8
3	M	60-79	COPD	Ipratropium bromide	SCC	Larynx	3/2/0		IV a	S + RT + CT	32,0
4	M	60-79	DM type II	Metformin hydrochloride	SCC	Larynx	4/3/0		IV b	S + RT + CT	33,9
5	M	60-79	None	None	SCC	Larynx	2/1/0		III	S + RT + CT	32,0
6	M	60-79	DM type II	Metformin hydrochloride, Atorvastatin calcium	SCC	Larynx	2/0/0		II	S + RT	31,5
7	F	40-59	Hypertension, hypothyroidism	Metoprolol, Levothyroxine sodium	SCC	Oropharynx	2/1/0		III	S + RT + CT	32,0
8	M	60-79	COPD	Ipratropium bromide	SCC	Oropharynx	2/3/0		IV b	S + RT + CT	31,5
9	F	40-59	None	None	SCC	Parotis	4/0/0		IV a	RT	32,0
10	M	40-59	None	None	Nonkeratinizing undifferentiated NPC	Nasopharynx	3/0/0		III	RT + CT	31,8
11	M	21-39	None	None	Nonkeratinizing undifferentiated NPC	Nasopharynx	2/2/0		III	RT + CT	31,8
12	M	40-59	DM type II	Metformin hydrochloride	Nonkeratinizing undifferentiated NPC	Nasopharynx	3/1/0		III	RT + CT	32,0
13	M	21-39	COPD	Ipratropium bromide	Keratinized NPC	Nasopharynx	2/2/0		III	RT + CT	33,9

Abbreviations: M, Male; F, Female; DM, Diabetes Mellitus; COPD, Chronic obstructive pulmonary disease; SCC, Squamous cell carcinoma; NPC, Nasopharyngeal carcinoma; S, Surgery; RT, Radiotherapy; CT, Chemotherapy.

**Table 2.** Staging and grading of periodontitis of the patients before HNC radiotherapy using the 2017 Classification of Periodontal Diseases.^19^

ID	Number of Teeth	Periodontitis Stage	Periodontitis Grade
1	14	IV	C
2	12	IV	C
3	11	IV	C
4	27	II	C
5	16	IV	C
6	20	IV	C
7	19	III	C
8	18	III	C
9	18	II	C
10	22	II	C
11	27	II	C
12	14	IV	C
13	26	II	C

The patients received curative cancer treatment and the average radiotherapy dosage of 32.3 Gy (SD = .91) during the first 3 weeks of radiotherapy. Nine patients received chemotherapy treatment in combination to radiotherapy. Concomitant chemotherapy was administered using Cisplatin. For patients with nasopharyngeal carcinoma neo-adjuvant chemotherapy (Gemcitabine, Cisplatin for three cycles) was used.

### Total Neutrophil Count, Neutrophil/lymphocyte Ratio

The total neutrophil count (NEUx1000) average before radiotherapy was 5.02 (SD = 1.04) and 4.51 (SD = 1.59) 3 weeks after radiotherapy started. A statistically significant difference was not observed despite seeing an average-based decrease in the total neutrophil count (P = .295).

The average neutrophil/lymphocyte ratio was 2.92 (SD = 1.16) before radiotherapy and 5.46 (SD = 2.49) 3 weeks after radiotherapy started. There was a statistically significant increase in the neutrophil/lymphocyte ratio, one of the indicators of systemic inflammation, 3 weeks after radiotherapy started (P = .005).

### aMMP-8 Analysis

**Figure 1.**
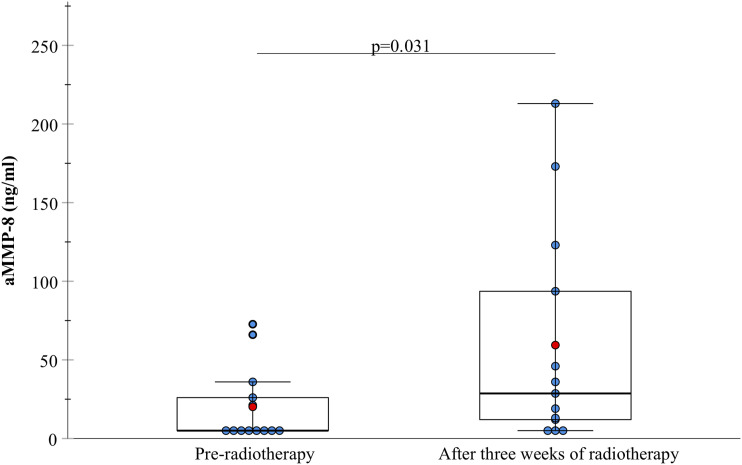
aMMP-8 level (ng/ml) before radiotherapy and 3 weeks after HNC radiotherapy started.

Three weeks of radiotherapy caused a statistically significant increase in the oral aMMP-8 levels (P = .031). Average aMMP-8 level of the study group was 20.1 ng/mL (SD = 24.1) before radiotherapy and 59.4 ng/mL (SD = 69.7) 3 weeks after radiotherapy started (Figure 1).

**Figure 2.**
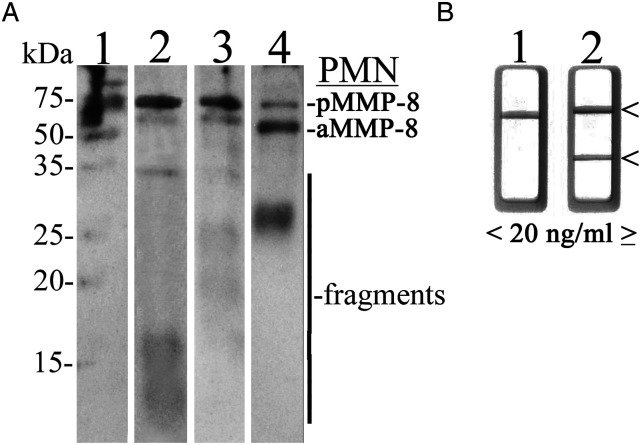
A: Representative western immunoblot analysis of MMP-8; B: chair-side (PoC) lateral flow immunotest. A: Representative western immunoblot for molecular forms and species of MMP-8/collagenase-2 in human mouthrinse and saliva. Lane 1: molecular weight standards; lane 2: recombinant human MMP-8, human salivary sample from periodontally and systemically healthy subject, polyclonal antibody; lane 3: human mouthrinse sample before radiotherapy, polyclonal antibody, lane 4: human mouthrinse sample 3 weeks after radiotherapy started, note the activation and fragmentation of MMP-8 to lower molecular weight species. The p and a indicate pro and active neutrophil (PMN) collagenase-2, respectively and fragments lower molecular size species. Molecular weight standards’ mobilities are indicated on the left. B: Negative (-, <20 ng/mL aMMP-8, lane 1) and positive (+, ≥20 ng/mL aMMP-8, lane 2) chair-side (PoC) lateral-flow immunotest outcomes indicated by arrows on the right.

Representative western immunoblot analysis of MMP-8 in the studied mouthrinse samples before and 3 weeks after radiotherapy started in HNC patients are shown in Figure 2. MMP-8 was converted to active and fragmented forms as analyzed by polyclonal anti-MMP-8 antibody.

## Discussion

While evidence exists regarding the destructive changes in periodontal tissues following HNC radiotherapy,^20,21^ it is not yet known which biomarkers have the best diagnostic value to predict and monitor the potential periodontal side effects in HNC radiotherapy.^22^ Significant increase in aMMP-8 levels, a potential indicator of periodontal destruction, 3 weeks after radiotherapy started was the main finding of the present pilot study.

The oral microbiota is known to be significantly changed during radiation therapy.^23^ It is suggested that radiotherapy creates a dysbiotic microbiota that changes the host metabolism provoking increased risk for inflammation^22^ and chemotherapy has been shown to decrease the microbiome diversity.^23^ It has been discussed that the alteration of the salivary bacterial flora caused by tumor itself or radiochemotherapy may contribute to mucosal damage, modulating the levels of opportunistic bacteria that can turn pathogenic in patients with HNC.^24^

In this study, we were interested in finding out the immediate effect of radiotherapy on periodontal health. After 3 weeks of radiotherapy, we can assume that the recorded changes in periodontal health parameters are caused by the radiotherapy as periodontitis derived from bacteria takes longer time than 3 weeks. Long-term side effects of the radiation therapy, such as dry mouth and possible motivational or physical challenges with dental self-care, have not yet significantly affected the oral health. Also, it was convenient to take mouth rinse samples as the patients came for a routine visit to the hospital.

The traditional periodontal examination methods such as measurements of gingival pocket depth, clinical attachment loss and bleeding on probing (BOP) as well as radiological examination evaluate already experienced tissue destruction. However, these examinations do not provide definitive information about the status of the disease (active or inactive) or the potentiality for future progression.^25^ Simultaneously, periodontal examination techniques such as BOP and probing depth are considered invasive methods and expose patients to bacteremia.^26^ Thus, in the present study it was decided to apply a non-invasive and quantitative chair-/bed-side method, the oral fluid aMMP-8 point-of-care test, to determine the periodontal degeneration. The immunological aMMP-8 test, that resembles classical pregnancy and COVID-19-antigen tests, provides information about active processes and phases of periodontitis. At the same time, it offers quantitative on-line and real-time values that has repeatedly shown positive results on periodontitis and dental peri-implantitis globally in several independent studies.^16,27^

It has been shown in some studies that periodontitis causes an increase in the total neutrophil count. According to the study by Pejcic et al among the individuals who have moderate to advanced periodontitis, the total neutrophil count is significantly higher than that in healthy individuals.^28^ Christan et al^29^ showed that periodontal treatment can cause reduction in the systemic neutrophil count. However, in this study statistically significant difference in the total neutrophil counts was not found 3 weeks after radiotherapy started. There was a significant increase in the neutrophil/lymphocyte ratio after 3 weeks of radiotherapy suggesting systemic subclinical inflammation. The significant increase observed in the aMMP-8 levels alongside the neutrophil/lymphocyte ratio can give an idea of the local damage that occurs in patients with HNCs receiving radiotherapy treatments. High neutrophil/lymphocyte ratios have been shown to associate with generalized aggressive periodontitis.^30^ Accordingly, it has been thought to be a potential indicator in identifying generalized aggressive periodontitis with increase in the systemic neutrophil/lymphocyte ratio. Similarly, in a study by Dogan et al,^31^ it has been observed that in individuals with periodontitis the systemic neutrophil/lymphocyte ratio increases. Therefore, the increased neutrophil/lymphocyte ratio could be regarded as a systemic risk factor for periodontitis.

aMMP-8 is a proteolytic enzyme that is mainly derived from neutrophils.^32^ Since aMMP-8 levels increased in the mouthrinse after 3 weeks of radiotherapy in this pilot study, in the absence of a concomitant change in the total neutrophil counts before and after radiotherapy, it can be speculated that radiotherapy eventually triggers or induces the neutrophils to degranulate, thereby activating the MMP-8, and increasing the risk of local damage in periodontal tissues.

It is known that the release and activation of MMP-8 is a complex process involving selective neutrophil subcellular granule content degranulation occurring as a result of complicated molecular mechanisms.^33^ More research is needed to better understand the impact of radiotherapy process on the periodontal pathogenesis in patients with HNC and the role of different aMMP-8 regulator molecules and processes such as TIMP-1, the main endogenous inhibitor of MMP-8 activation.^34^

The findings of this pilot study must be interpreted with the following limitations. Significant confounding features are small study group, variations in HNC locations and treatments and additional chemotherapy for portion of the patients. For all seven patients who underwent surgery, the radiotherapy was given post-operatively. Performed surgery might as well have an effect to inflammatory markers with radiotherapy. The radiation is targeted to the tumor location, so there’s a different amount of radiation to the jaws and periodontium, depending on the HNC radiation field. The majority of the cases evaluated were of laryngeal or nasopharyngeal primary, which are typically associated with lesser oral cavity radiation dose compared to oral cavity or oropharyngeal primaries. However, they all had a head and neck carcinoma and the radiotherapy had effects on oral region although the radiation dosage was within close limits. aMMP-8 oral rinse test evaluates the situation in the oral cavity which allows some variation since it is not site specific. Oral mucositis is an acute tissue injury after HNC radiotherapy and majority of the patients develop some degree of mucositis.^35^ In patients receiving a typical 6-7 week course of RT, oral mucositis presents as mucosal erythema during the first 2-3 weeks of RT. As the dose of radiotherapy increases, oral mucositis progresses to ulceration and pseudomembranes.^35^ The results of our study reveal worsening of oral immunity following radiotherapy in HNC patients and we encourage further research on the potential benefit of aMMP-8 point-of-care tests for screening and monitoring of the real-time periodontal health-status for patients’ undergoing cancer treatments. We have previously shown a similar significant positive correlation between aMMP-8 levels (both pre- and post-radiotherapy) and the difference between the mean of probing depths pre-and post-radiotherapy.^36^

We suggest that the health of the periodontium may be weakened by the direct side effects of the radiotherapy on the periodontium (e.g. hypovascular, hypocellular and hypoxic changes), alteration of the oral microbiota during HNC (chemo)radiotherapy and underlying pre-existing periodontitis. All these may change the host metabolism and provoke inflammation.

## Appendix

### Notation

MMP-8: Active-Matrix Metalloproteinase-8
HNC: Head and Neck Carcinoma
NPC: Nasopharyngeal Carcinoma
POC: Point-Of-Care
RT: Radiotherapy
SCC: Squamous Cell Carcinoma
